# Development of a new reproductive tissue cryopreservation clinical service for children: the Oxford programme

**DOI:** 10.1007/s00383-019-04503-3

**Published:** 2019-07-02

**Authors:** K. Lakhoo, J. Davies, S. Chakraborty, S. Berg, R. Tennyson, D. Fowler, S. Manek, C. Verrill, S. Lane

**Affiliations:** 1 Department of Paediatric Surgery, University of Oxford and Oxford University Hospitals, Oxford, UK; 2 Oxford Tissue Bank, University of Oxford and Oxford University Hospitals, Oxford, UK; 3 Department of Paediatric Radiology, University of Oxford and Oxford University Hospitals, Oxford, UK; 4 Department of Paediatric Anaesthesia, University of Oxford and Oxford University Hospitals, Oxford, UK; 5 Department of Psychology, University of Oxford and Oxford University Hospitals, Oxford, UK; 6 Department of Cellular Pathology, University of Oxford and Oxford University Hospitals, Oxford, UK; 7 Department of Paediatrics and Child Health, University of Oxford and Oxford University Hospitals, Oxford, UK; 8 Nuffield Department of Surgery, Oxford University and Oxford University Hospitals, Headley Way, Oxford, OX39DA UK

**Keywords:** Children, Young adults, Cryopreservation, Ovary, Testis, Reproductive tissue, Premature ovarian and testicular insufficiency

## Abstract

**Purpose:**

This article describes the development of a new reproductive tissue cryopreservation clinical service for children at high risk of infertility in the NHS during times of severe financial constraints in the health service.

**Method:**

A development plan with two phases was drawn up. Phase 1 restricted the service to childhood cancer patients referred to the Oxford Paediatric Oncology and Haematology Principle Treatment Centre. It was estimated that there would be 10 patients/year and used existing staff and facilities from paediatric oncology, surgery, anaesthetics radiology, pathology, psychology, teenage–young adult gynaecology, and an existing Human Tissue Authority tissue bank with a licence for storage of tissue under a Human Sector Licence. Phase 2 extended the service to include children and young adults across England, Wales and Ireland—patients from Scotland having access to a research programme in Edinburgh. The main challenge in phase 2 being resources and the need for patients to be able to be treated as close to home as safely as possible.

**Results:**

The Oxford team developed information resources and eligibility criteria based on published best practice, referral and treatment pathways, multidisciplinary team meetings, a network of third party sites, and a dedicated case management and database. As the programme expanded, the Oxford team was able to justify to management the need for a dedicated theatre list. Patient feedback through questionnaires, qualitative work conducted as part of a Ph.D. thesis as well as direct patient stories and interviews in TV, and radio features underpins the positive impact the programme has on patients and their families.

**Conclusion:**

The Oxford Reproductive Cryopreservation programme delivers fertility preservation treatment to children and young adults at high risk of infertility safely, effectively and as close to home as possible. The onward view is to apply for national funding for this programme for recognition and sustainability.

## Introduction

Fertility is the ability to have babies or to reproduce. Very early loss of fertility can occur for a number of reasons both in children and young adults. Premature loss of fertility is the single most feared long-term consequence of childhood cancer survivors and leads to significant morbidity [1, 2]. Survivors of childhood cancer have been shown to be 40% less likely than their matched sibling pair to have a family [3]. With cure rates for childhood cancer above 85%, there is an increasing cohort of long-term survivors of childhood cancer for whom fertility is an important consideration.

There are fertility preservation options for patients who are physically and psychologically old enough and have sufficient time pre-cancer treatment to store eggs/embryos or sperm. For those too young or where treatment is required very urgently alternative treatment options are required. Reproductive tissue cryopreservation, which enables immature eggs in girls and spermatagonial stem cells in boys to be preserved, is now possible [4, 5].

Over the last 20 years a number of International Centres have set up reproductive tissue cryopreservation programmes. Initially these programmes were research focused but as outcome data have become available, showing success rates as measured by live births, similar to other forms of fertility preservation so the emphasis has moved to providing reproductive tissue cryopreservation as a clinical service [6]. The latest published outcome data from 2018, documents around 150 live births from cryopreserved ovarian tissue that has been auto-transplanted. Clinical programmes offering reproductive tissue cryopreservation have been established in a number of countries. In the UK, Edinburghhave had a research programme running since 1993, but until the Oxford programme was launched in 2013 there was no clinical service available to patients in England. In England, reproductive tissue storage can only be performed by licensed tissue banks which comply with European Tissue and Cell Directives and UK Quality and Safety Regulations (Q & S regulations) [7]. The activity is licenced and regulated in England by the Human Tissue Authority (HTA).

Establishing children and young adult fertility services including procurement and storage of reproductive tissue from children and young adults many of whom have just received a diagnosis of cancer are acutely unwell and need very urgent intervention is challenging. It has required coordinated, collaborative working across a number of different teams, excellent communication, clear referral criteria, and well established patient treatment pathways with real time review by a multidisciplinary team of referrals that fall outside the agreed eligibility criteria.

The National Institute for Health and Care Excellence (NICE) fertility guidelines for patients diagnosed with cancer directs all the health care professionals to discuss the effect of proposed disease treatment on a patient’s future fertility and to offer fertility preservation treatment where appropriate [8]. The Oxford Reproductive Tissue Cryopreservation Programme makes it possible for professionals to discuss fertility risks and options with patients of all ages.

## Oxford children and young adult fertility service

In 2008, Oxford University Hospitals NHS Foundation Trust in collaboration with the University of Oxford Department of Women and Reproductive Health embarked upon a programme to develop and introduce reproductive tissue cryopreservation. Initially it was not clear whether the governance and licencing of such a service would fall under the Human Tissue Authority (HTA) or the Human Fertilisation and Embryology Authority (HFEA). The Oxford programme obtained and maintains licences from both competent authorities although subsequently it has become clear that reproductive tissue stored for the purpose of auto-transplantation fall under the governance and licencing of the HTA. To meet the HTA requirements, the Oxford team worked in close collaboration with Professor Ariel Revel and his team at Hadassah University Hospital. Professor Revel’s team have had a successful programme running for a number of years with live births from stored tissue that has subsequently been auto-transplanted tissue. The Oxford team continues to collaborate with Professor Revel with exchange visits to benchmark the programme. Viability of the stored tissue has been documented using formal histopathology and tissue culture techniques.

**Fig. 1:**
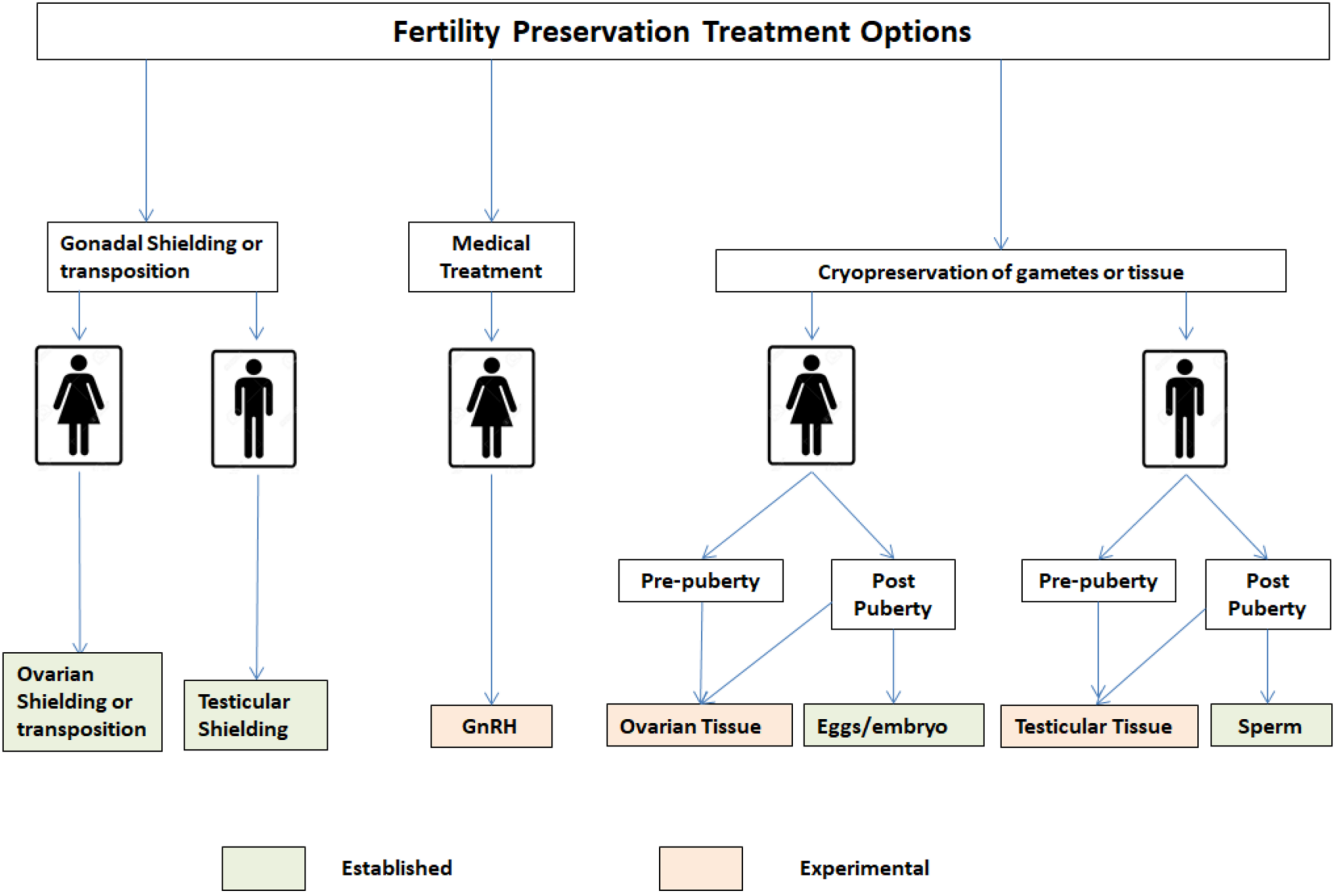
Fertility preservation treatment options

In 2013, the Oxford Children and Young Adult Fertility Preservation Service was launched with a mission to provide information, advice and fertility preservation treatment where appropriate to children and young adults at the risk of premature loss of reproductive function. To help facilitate discussions about the effects of chemotherapy and radiotherapy on fertility and fertility preservation treatment options, the Oxford Children and Young Adult Fertility Service developed information leaflets, a website, and online video resources. Clinicians are encouraged to seek advice about fertility options available for their patients and where appropriate the patient and/or family can have a face to face or telephone consultation to help inform them about risks and benefits of fertility preservation treatment options. Treatment options available to patients are summarised in Fig. 1.

Ovarian and Testicular tissue cryopreservation treatment is still relatively new technology and is currently classified as experimental. The term ‘experimental’, as defined by the Medical Research Council (MRC), reflects current practice and acknowledges that tissue can be procured and stored successfully, but methods of clinical use are still evolving. A critical step in the development of the Oxford programme was definition of the eligibility criteria. We based the criteria on those used by other major centres around the world and those published by Anderson and Wallace [9]. The eligibility criteria used in the Oxford Programme are stated in Table 1.

**Table 1:** Eligibility criteria

Female	Male
Under 35 years of age and unable to store eggs	Pre- pubertal or unable to store sperm
High or at very high risk of infertility*	High or at very high risk of infertility*
Oncology patients must be being treated with curative intent	Oncology patients must be being treated with curative intent
Medically fit for fertility preservation treatment	Medically fit for fertility preservation treatment
Fertility treatment must not delay primary treatment	Fertility treatment must not delay primary treatment

*As defined by published data (Ref Children’s Cancer and Leukaemia Group Fertility Consensus document)

Development of a secure referral process and definition of the treatment pathway were further critical steps. Referrals to the Oxford programme must be made by submission of a standard referral form available from the service coordinator. Information submitted on the form is then reviewed by the Programme Clinical Lead or Deputy. If the referral is within eligibility criteria the patient is accepted and scheduled for surgery to fit with timing of their primary treatment. If the referral falls outside criteria it is referred to the Programme Multidisciplinary Team (MDT) for discussion and decision about the fertility treatment. The programme MDT includes a Paediatric oncologist, Paediatric surgeon, teenage, and young adults (TYA) Gynaecologist, Gynaecology–Fertility Experts, Paediatric anaesthetist, the HTA and HFEA license holders (the Biobank lead) and research leads for ovarian and testicular work. The MDT has access to a Consultant Ethicist when required. The MDT meets formally once a month and in addition on an ad hoc basis as required by the clinical need.

A service coordinator for the programme is essential coordinating contact with the patient and family, referring centre, arranging admissions, theatre, and all relevant clinical and laboratory teams. The nature of the programme means that patients need treatment urgently and the service can be very fluid. Ensuring the highest level of coordination in the ever changing landscape is crucial to success.

Prior to surgery, the Service Clinical Lead or deputy will to meet with the patient/family either face to face or via a Skype or telephone conference call to ensure that the patient and, if appropriate, family have all available information and have an opportunity to ask questions to enable them to give informed consent. The discussion must ensure that the patient/family has accurate information and realistic expectations as to what is currently possible so as not to raise false hope and expectations for the future.

In accordance with the HTA licence requirements, patients and/or their parents/guardians must sign a specific tissue consent form. The consent form details the licence holders responsibilities to the individual, outlines the right to withdraw consent at any stage, and details the patient’s/parents/guardians wishes for use/disposal of any tissue that is either too small to freeze or not required which includes the use/disposal of tissue should the individual die. For children under the age of 18 years, the parents/guardians give written consent. All children under the age of 18 years will need to re-consent for continued storage and disposal of tissue when they reach the age of 18 years.

Tissue that is too small to freeze or not required by the patient can either be disposed of as per hospital policy for disposal of tissue or donated for research. Tissue donated for research will be held in the biobank and will be anonymised prior to release for use in research programmes with research and development approval. The research programmes must be designed to improve fertility preservation treatment for children and young adults.

Patients are admitted for surgery under the care of the surgeon performing the tissue procurement. It is the responsibility of the admitting surgical team in coordination with the referring centre/team to ensure the patient is fit for surgery. Many patients will need full medical assessment prior to surgery. This requires coordination between the surgical team, referring medical team, consultant anaesthetist, and where blood products are required the blood bank as often patients require very specialised blood products. The patients have often been recently diagnosed with malignant disease and it is critical that their care is appropriately managed. They often require isolation/cubicle care as they have poor immunity and bone marrow function. On discharge it is essential that follow-up arrangements are made with the referring hospital with full details of the procedure and follow-up care required.

In the first phase of the Oxford programme all patients having reproductive tissue procurement came to Oxford for the surgical procedure. To provide fertility preservation to patients across England a ‘Hub and spoke’ model has been developed which allows patients to have procurement as close to home as possible in what is termed a ‘spoke site’, where they are looked after by their local team. The tissue is then transported by a dedicated courier to the ‘Oxford Hub’ for processing and storage. In this model, which has been developed with extensive discussion with the HTA, the procurement at the ‘spoke site’ is conducted under a third party agreement to the Oxford HTA Licence. The third party agreement defines the responsibilities delegated to the spoke and those retained by the Hub. A third party site must be able to demonstrate that they have appropriately qualified medical and surgical staff, theatre facilities that meet licencing requirements and patient pathways to ensure compliance with the Hub-HTA licence. This model of service delivery has enabled patients across England to have equitable access to reproductive tissue cryopreservation.

## Anaesthetics

A comprehensive pre-operative assessment together with a fully informed consent is crucial for this group of patients [10]. The anatomical and physiological effects of cancer and the associated complications of chemotherapeutic agents are important considerations. Of relevance to the anaesthetist is that chemotherapeutic agents are associated with cardiotoxicity including cardiomyopathy and conduction defects; pulmonary toxicity including interstitial pneumonitis, pulmonary fibrosis, and pleural effusion; acute renal toxicity; nausea, vomiting, malnutrition, mucositis, and neurotoxicity. Anaemia, thrombocytopenia, and immunosuppression are also common problems. Haemoglobinopathy patients with a chronic blood transfusion regime can present with left ventricular dysfunction, arrhythmias, pulmonary hypertension, and valvular abnormalities, and will require a full cardiology assessment. These patients need to be fully assessed at their referring hospitals. Good communication and liaison between the centres is pivotal to ensure a smooth pathway and avoid the possibility of cancellation. Patients with very high-risk airways that occur in certain metabolic conditions will need to have procedures undertaken at designated centres, where these types of anaesthesia are regularly undertaken.

There are significant psychological factors for children undergoing treatment for cancer. Pain relief is paramount. Many children report that the most unpleasant part of their treatment was a painful medical or surgical procedure. They may have required opioids previously and opioid tolerance can be an issue. Anaesthetic responsibility includes providing a calm reassuring manner and assiduous management of pain, nausea, and vomiting. Pain management employs a multimodal approach.

Prophylactic antibiotics are routinely given at induction, and two blood samples are taken for mandatory serology testing and infectious disease markers.

General anaesthesia for ovarian cryopreservation requires tracheal intubation and ventilation. Post-operative pain relief can be challenging. Single incision laparoscopic surgery (SILS) is more painful than the three-port laparoscopic surgery in the immediate phase [11] and there is a proportionally greater stretching of the peritoneum compared to adults. The analgesic strategy includes bilateral rectus sheath block under ultrasound guidance, local infiltration at the port site, intravenous or oral opioids, NSAIDS, and paracetamol.

General anaesthesia for testicular biopsy utilises a supraglottic airway (SAD) and a spontaneous ventilation technique. This again can present challenges in the pain management. In children < 20 kg, a caudal extradural block is very effective. In older children, a spermatic cord block performed by the surgeon under direct vision plus local infiltration to the scrotal skin incision usually works well in addition to NSAIDS and paracetamol.

In both procedures, simple post-operative analgesics should be prescribed regularly rather than PRN. Using these multimodal techniques most children can be managed as day cases providing that they fulfil the prerequisite criteria [12].

## Surgery and radiology

Starting a new service in paediatric surgery without extra resources inevitably has an impact on the general service; however, evidence that a new service is both possible to deliver and needed requires a ‘trial’ period. The surgical resource issue was addressed in Oxford by backfilling lists unfilled due to annual and study leave and used the supportive/administrative programmed activity (SPA) time of the senior staff to develop the surgical component of the service. After 5 years of running the programme as above, the national and international use of the service showed financial gain to the institution and so it was decided that there should be a dedicated weekly theatre list. This was a very important step as it has allowed a dedicated team of surgeons, anaesthetists, theatre staff and support teams to be developed to provide directed care for patients having reproductive tissue cryopreservation.

The patients are discussed and referred to the lead paediatric surgeon for this service. Patients admitted for surgery are under the care of the admitting surgical team who are responsible for ensuring the patient is fit for the designated surgery and for follow up care in the post-operative period. Surgical consent is taken for the oophorectomy or testicular biopsy and any associated procedures such as insertion of central venous line/gastrostomy. Most patients have a concurrent procedure of central line, bone marrow aspirates, feeding gastrostomy etc. which must be clearly specified on the theatre list. It is imperative that referring teams specify whether a double or triple line is required and give a clear history of previous line insertions as this may be challenging if venous access is compromised. Line service is provided by the paediatric radiology department using a percutaneous route for line insertion. Generic double and triple lumen lines are available, but if special line is required this is supplied by the referring team.

For the testicular biopsy, the size of both the testis is measured in the theatre before the procedure commences using an orchidometer and the right testis is biopsied unless the right testis is not of a suitable size. The size of the testis must be recorded by the attending tissue bank technician. The scrotal skin is opened using a transverse incision, testis delivered, hydatid if present excised, and a pole to pole 33% wedge biopsy taken. The biopsy specimen is transferred into the container provided by the tissue bank technician who is present in the theatre to collect the sample in accordance with Q & S regulations.

For the ovary, single port laparoscopic technique is used. The umbilicus is cleaned, local anaesthetic injected if no bilateral rectus sheath block is given. A vertical incision is performed through the umbilicus. Gelpoint Mini Advanced Access platform^®^ (Applied Medical) device is used which has an Alexis and 3-port flexible gel cap. The ovaries are both visualised and photographed and the right oophorectomy was performed using a ligasure device^®^ (Covedien). Left oophorectomy is only performed if the right ovary is not suitable or the patient is due to have radiotherapy that will include the left ovary. The gelpoint structure is decapped to remove the ovary which is placed directly into the specimen pot provided by the tissue bank technician. Intravenous or oral analgesia is used and most cases are discharged on the day unless the co-morbidities of the disease or analgesia control require overnight stay or the location of the patient is beyond our regional referral zone. After 2 years of service, we now have a weekly dedicated list for cryopreservation which has provided easier co-ordination and predictability of the timing of surgery with the tissue bank for safe retrieval, processing, and storage of tissue.

From 2013 to 2018, we received over 700 referrals of which 513 proceeded to storage of reproductive tissue. Of the 513 samples procured, 362 were ovarian and 151 testicular. The Oxford Reproductive Tissue Cryopreservation Programme (ORTCP) receives enquiries and referrals from clinicians across England, Wales, and Ireland. There are a number of reasons why referrals do not proceed to procurement. These are summarised below:

- Patients do not fulfil eligibility requirements and after discussion at MDT and then subsequent discussion with the referring consultant the patient does not proceed to tissue storage.
- Patients after referral have disease progression or are medically unfit for surgery and so cannot proceed to tissue collection.
- Patients referred who have a diagnosis of Hodgkin’s lymphoma who will receive initial non-gonadotoxic treatment only proceed to tissue collection if they need to escalate to more toxic therapy.
- Patients or their parents/guardian may withdraw after treatment/consent consultation as they decide on a risk–benefit analysis that they wish to proceed to definitive treatment without fertility preservation treatment.

There were 262 (51.1%) cases with underlying malignant disease and 100 (19.5%) with benign disease where ovarian tissue was procured, and 75 (14.6%) cases with malignant disease and 76 (14.8%) benign disease where testicular tissue was procured.

There have been no serious adverse events reported. Adverse events/morbidity from the procedure is very low with one umbilical wound infection, one umbilical hernia, and three scrotal haematomas. The overall morbidity was 0.8%. None of the adverse events led to a delay in definitive treatment.

## The role of the tissue bank

To preserve ovarian and testicular tissue intended for transplantation (‘human application’), Oxford had to ensure compliance with Q & S regulations [13], the Human Tissue Act 2004 [14], and are required to meet the standards which are detailed in the Guide to Quality and Safety Assurance of Human Tissues and Cells for Patient Treatment [7] and the Human tissue authority (HTA) Codes of practice [15]. This was achieved by recruiting the services of the existing licensed tissue bank, the ‘Oxford Cell & Tissue Biobank’ (OCTB) which already had the staff, premises, equipment and the relevant knowledge and understanding. Since the intention from the outset was to start the first clinical service, after liaison with the UK regulatory authorities for tissues and gametes (HTA and HFEA), OCTB completed extensive validation (using animal and human tissue) prior to submitting a preparation process dossiers (PPDs) based upon the adopted protocols used successfully at Hadassah University Hospital, Israel. Hadassah also very generously shared their experience and provided training for OCTB staff. Under the terms of the Q & S regulations, OCTB was subsequently awarded licenses from HTA and HFEA, and the clinical service was commenced in September 2013 [16]. As the service developed, OCTB gained approval from HTA to procure tissue at third party centres under the OCTB license following implementation of third party agreements. On the day of surgery, OCTB technician must attend procurement to ensure consent is checked, tissue is collected with minimal risk of microbiological contamination, and to perform required witnessing, labelling, and packaging of the tissue and blood sample before transporting in validated containers back to the OCTB cleanroom facility for processing.

Tissue is then registered at OCTB and a unique code number is allocated. At all stages of processing, witnessing by two technicians has to be employed with a detailed system of documentation to facilitate traceability, ensuring a robust audit trail is maintained. The tissue is transferred into the OCTB cleanroom facility which meets grade A air quality requirements [17] and is then dissected to produce cortical tissue standard sized < 2 mm thick sections. After adding optimised cryoprotectants and slow controlled freezing process (“cryopreservation”), tissue is finally transferred to long-term storage at < − 170 °C in vapour-phase liquid nitrogen at OCTB. A tissue segment is concurrently sent for histological examination and microbial sampling of all in-process fluid samples is performed according to European Pharmacopeia [18] and SaBTO requirements [19]. The cost of processing and storage of reproductive tissue is funded through a dedicated charitable fund as part of the Oxford hospitals charity and underwritten by the Oxford University Hospitals NHS Foundation Trust as part of the requirements of the HTA licence.

At the age of 18 years the patient is reconsented for continued storage of their tissue.

If the autologous transplantation is agreed by all relevant parties, the patient has to give consent for a specific amount of tissue to be thawed for use and the required risk assessment has to be completed by OCTB. Any tissue which is not used in the re-implantation surgery will be returned directly to OCTB to be registered and then sensitively disposed according to OUHFT standard procedure. Tissue will also be removed from OCTB storage and disposed after a patient death has been confirmed and when there is no consent for research use.

UK regulatory authorities are required to inspect licensed premises in the human application sector at least every 2 years. OCTB has most recently successfully undergone on-site inspection in February and March 2019, all reports are published [20].

## Pathology

Most young patients who undergo cryopreservation to preserve fertility potential have been diagnosed with malignancy or having a bone marrow transplant for bone marrow failure syndromes or haemoglobinopathies. In some patients they may have had exposure to chemotherapy prior to tissue collection. It is known that, spermatogonial quantity is significantly reduced in testes of prepubertal boys treated with alkylating agents and other gonadotoxins [21], and so pathology review with documentation of tissue quality, presence of spermatagonial stem cells, and any histological evidence of malignancy is essential. Similarly for girls and young women who are at high risk of premature ovarian insufficiency and infertility, storage of ovarian tissue and documentation of tissue quality, presence of primordial follicles, and evidence of malignancy is essential [22].

As in most clinical situations the main role for the pathologist is to exclude malignancy, document whether the gonadal tissue looks normal, or not, and assess the presence or absence of spermatogonia or primordial follicles. This assessment can be semi-quantitative or qualitative (absence/presence). Several methods have been used, for example, the number of spermatogonia per transverse tubular cross-section (S/T). Other workers have used immunomarkers like placental alkaline phosphatase (PLAP) positive gonocytes in testes within year 1 of life (in the clinical setting of bilateral cryptorchidism) to indicate preserved ability for germ cell transformation [21–24].

The tissue samples are pre-freeze samples of variable quantity, ovary samples are usually 5–10 mm in length and 1–2 mm depth, testis samples are usually smaller, 1–5 mm length; the samples are entirely submitted for histology. The tissue is processed, embedded in paraffin; 4 micron sections are cut and stained with H&E in the standard way that all UK NHS clinical laboratories use. Some workers have used semi-thin (< 2 micron) sections stained with toluidine blue to improve cellular characterisation but at present we do not employ this technic in Oxford.

## Psychology

The role of the Clinical Psychologist in the team is to offer support and counselling to patients if required. It is also important to understand the impact new treatments have on thepatients. We have carried out qualitative research exploring the meaning of undergoing ovarian tissue cryopreservation in teenagers and young adults with cancer. A study was undertaken and the results formed part of a doctoral theses and a manuscript for publication is in preparation. In the pilot study, eight females who had undergone the procedure at Oxford University Hospitals NHS Foundation Trust were interviewed by a researcher who was independent to the clinical service. Key messages from participants pointed to the importance of having access to this procedure with relation to three key themes: the meaning associated with having the procedure, the role of the procedure in promoting positive adjustment to the cancer diagnosis, and the longer term impact of having the procedure on reducing the longer term negative impacts of having cancer. Having the option of the procedure was reported to foster a sense of hope; not just the chance of being able to conceive in the future, but of implied survival from the disease. Other positive effects included a sense of having more control over life post cancer, having a wider range of options than previously thought, and a sense of feeling lucky. All these factors were perceived by participants to be positively impacting on their psychological wellbeing.

These findings are particularly important given that previous research indicates that infertility can be more difficult to come to terms with than the longer term impact of the diagnosis of cancer itself, and that poor adjustment to a health condition is linked to higher incidences of anxiety and depression, which in turn impacts on future health behaviours. The detailed and rich accounts of the importance of having access to the procedure provide compelling evidence that its availability contributes positively and significantly to adjustment to the cancer diagnosis and the long-term psychological impact of the undergoing treatment. Therefore, the significance of having a fertility preservation option available to teenagers and young women cannot be understated.

## Summary

We now have a fully intergrated clinical service with biweekly children and young adult fertility clinic, hub, and spoke national referral programme with third party agreement in place, over 700 referrals and 500 tissue specimen stored.

We have a HTA licensed biobank, and have sucessfully retained HTA and HFEA licences since 2013 for collection and storage of ovarian and testicular tissue, and have upgraded our tissue storage facility to a state of the art 24 h computerised monitoring facility.

We have a national and international research collaborative programme and hope to achieve central NHS funding to sustain this programme.
